# Predictive determinants of scorpion stings in a tropical zone of south Iran: use of mixed seasonal autoregressive moving average model

**DOI:** 10.1186/s40409-017-0129-4

**Published:** 2017-08-23

**Authors:** Vahid Ebrahimi, Esmael Hamdami, Mohammad Djaefar Moemenbellah-Fard, Shahrokh Ezzatzadegan Jahromi

**Affiliations:** 1 Nephro-Urology Research Center, Shiraz University of Medical Sciences, Shiraz, Iran; 2 Social Determinants in Health Promotion Research Center, Hormozgan Health Institute, Hormozgan University of Medical Sciences, Bandar Abbas, 79391-83417 Iran; 3 Research Center for Health Sciences, Institute of Health, Department of Medical Entomology and Vector Control, School of Health, Shiraz University of Medical Sciences, Shiraz, 71645 Iran

**Keywords:** Climate, Mixed seasonal ARMA, Regression analysis, Scorpion, Scorpion stings, Time series, Iran

## Abstract

**Background:**

More than 1.2 million scorpion stings occur annually worldwide, particularly in tropical regions. In the absence of proper medical care, mortality due to venomous scorpion stings is an important public health issue. The aim of the present study is to explore the temporal trend of scorpionism with time series models and determine the effective factors on this event using regression models.

**Methods:**

A retrospective cross sectional study was conducted on 853 scorpion stung patients. They were referred to Haji-Abad Hospital of Hormozgan University of Medical Sciences (HUMS), south Iran, from May 2012 to July 2016. A linear model to describe and predict the monthly trend of scorpion sting cases is fit with autoregressive moving average (ARMA) model.

**Results:**

Of 853 victims, 384 (45%) patients were female and 30.2% of them lived in urban areas. The mean (± SD) age of patients was 30.1 (± 19.6) years and the most affected age group was 20-29 years (21.8%). Most victims were unemployed people and farmers (54.7%) followed by housewives (30.2%). The majority of the stings occurred indoors (53.7%), between midnight and 6 a.m. (29.2%), in the summer (44.2%), and the most affected limbs were hands and legs (81.2%). Patient genders and occasions of being stung by scorpions were significantly different between outdoors and indoors (*p* < 0.001). Scorpion stings due to *Odontobuthus doriae* were significantly higher than due to other species in urban and rural patients (*p* = 0.04). Mixed seasonal ARMA at lag 12, ARMA (1, 1) × (0, 1), was selected as the best process for monthly trend of data. Regression results indicated that significant climate factors associated with scorpion stings are temperature (*p* < 0.001) and relative humidity (*p* = 0.002).

**Conclusions:**

Scorpion stings have a noticeable effect on tropical rural populations, mainly farmers. Two effective climate factors associated positively and negatively with scorpion sting cases are temperature and relative humidity, respectively. The results of time series and regression models to predict the trends and determinants of scorpion stings are almost the same.

## Background

There are more than 1500 different species of scorpions in the world and only about 50 of them are medically important to humans [1]. The most dangerous scorpions are found in South America, North Africa, South Africa, Middle East, and India [2].

Scorpions are potentially fatal venomous arthropods with nocturnal habits that rest in shelters during the day. Their venoms – composed of low-molecular-weight neurotoxic peptides with lethal and crippling effects – are injected into the victims via a sharp sting at the end of their tails [3, 4]. Most scorpion venoms destroy red blood cells and cause painful swelling at the sting site [3, 5].

Depending on the scorpion species, the victim can be dead in less than seven hours [6]. Globally speaking, the mortality rate due to the scorpion stings is 0.27% [7]. The two main variables that affect the severity of scorpionism are: the characteristics of the victim (such as age and health condition) and the characteristics of the scorpion (such as species and venom potency).

Despite abundant studies on scorpions worldwide, the actual incidence of scorpion stings in some areas is not clear. Nevertheless, the average incidence of scorpion stings is estimated to be about 1.2 million per year in the world [2]. The diversity of scorpion species is increased in tropical regions in latitudes between 23 and 38 degrees [8]. Given the geographical coordinates of Iran (between 25 and 40 degrees north), the scorpion distribution and species diversity in the country are remarkable [9, 10].

The incidence of scorpion stings in tropical and subtropical regions is greater than in other regions. After Mexico, Iran has the highest rate of the scorpion stings in the globe [11]. The majority of the stings that occur in the country are reported from the province of Khuzestan, followed by Sistan-Baluchistan, and Hormozgan [9]. About 50 species of scorpions are found throughout the territory and distributed into four families: Diplocentridae, Buthidae, Scorpionidae, and Hemiscorpiidae. Most Iranian venomous scorpions belong to the large family Buthidae, which is dangerous and mostly found in tropical and subtropical regions [12]. The recorded scorpionism cases in the country had been estimated to be between 40,000-50,000 per year, and despite treatment approximately 20 people die every year [2].

The study of scorpion fauna and the epidemiology of their stings have indicated that at least twenty species of scorpions from three families – namely Buthidae, Scorpionidae, and Liocheliade –were identified in Hormozgan province. *Odontobuthus doriae* of the Buthidae family appears to be the dominant species [13]. Distribution of most common species of scorpions in Hormozgan province is shown in Table 1. The species *Hemiscorpius lepturus* of the family Liochelidae is the most dangerous in Iran and also the one involved in most cases of mortality in Hormozgan province [13]. From 2011 to 2014 at least 2300 cases of scorpion stings were recorded in this province, causing the death of four children [13]. In order to forecast the future trend of scorpion stings in this area and adopt the indispensable measures to ameliorate such problem, statistical analyses (such as time series processes) could be implemented to provide a foundation to logical decision making.

**Table 1:** Distribution of common species of scorpions in Hormozgan province, south Iran

Family	Genus	Species	Size (mm)	Iran distribution	World distribution
Scorpionidae	*Scorpio*	*S. maurus*	65	Azerbaijan, Bushehr, Chahar-Mahal Bakhtiyari, Isfahan, Fars, Gilan, Hormozgan, Ilam, Khorasan, Khuzestan, Kohgilouyeh Boyer-Ahmad, Kurdestan, Lorestan, Qazvin, Semnan	North Africa: Algeria, Egypt, Libya, Mauritania, Morocco, Senegal, Tunisia; Asia: Iran, Iraq, Israel, Jordan, Kuwait, Lebanon, Qatar, Saudi Arabia, Syria, Turkey, Yemen
Buthidae	*Odontobuthus*	*O. doriae*	50	Found at high elevations of west, southeast, and central parts of Iran	Endemic to Iran
	*Mesobuthus*	*M. eupeus*	60	Ardebil, Azerbaijan, Isfahan, Golestan, Hormozgan, Kerman, Khorasan, Markazi, Mazandaran, Semnan, Sistan Baluchistan, Tehran, and Yazd	Afghanistan, Armenia, Central Asia, China, Georgia, Iran, Iraq, Turkey
	*Androctonus*	*A. crassicauda*	120	Throughout Iran	Iran, North Africa, West Asia
	*Orthochirus*	*O. scrobiculosus*	30	Throughout Iran	Central Asia, Iraq, Jordan, Iran, south Israel
	*Hottentotta*	*H. saulcyi*	60–100	Chahar-Mahal Bakhtiyari, Hamadan, Hormozgan, Ilam, Kermanshah, Khuzestan, Kohgilouyeh va Boyer-Ahmad, and Lorestan	Afghanistan, Iran, Iraq, Turkey
		*H. jayakari*	65–90	Hormozgan	India, Iran, Oman, Saudi Arabia, United Arab Emirates, Yemen
	*Compsobuthus*	*C. matthiesseni*	30–45	Azerbaijan, Bushehr, Chahar Mahal Bakhtiyari, Fars, Hamadan, Ilam, Kerman, Kermanshah, Khuzestan, Kohgilouyeh Boyer-Ahmad, Kurdestan, Lorestan, Markazi, Qom, Hormozgan	Iran, Iraq, Syria, Turkey
	*Simonoides*	*S. farzanpayi*	30	Hormozgan, Kerman, Sistan Baluchistan	Afghanistan, Iran, Pakistan
Liochelidae	*Hemiscorpius*	*H. lepturus*	50–75	Isfahan, Fars, Hamadan, Hormozgan, Kohgilouyeh Boyer-Ahmad, Kerman, Khuzestan, Lorestan	Iran, Iraq

The current dataset comprises time series data, that is, the data obtained from the observation of a phenomenon over time. Various processes including autoregressive (AR), moving average (MA), and mixed seasonal auto-regressive moving average (ARMA) can be used to model time series. Each of these models contains a set of processes with different parameters which could be applied as possible and suitable options in modeling [14, 15].

Therefore, the present study aims to:

- describe demographic and epidemiologic features of patients affected by scorpion envenomation in Haji-Abad, south Iran;
- analyze the trends of scorpion stings during 51 months, from May 2012 to July 2016, and look for peaks and troughs during this time period;
- observe what climatic factors are associated with the activity of scorpions in tropical areas of south Iran; and
- estimate the amount of antivenom that must be available for victims per year by predicting the number of scorpion stings using mixed seasonal ARMA and multiple regression models.

## Methods

### Study area and population

**Fig. 1:**
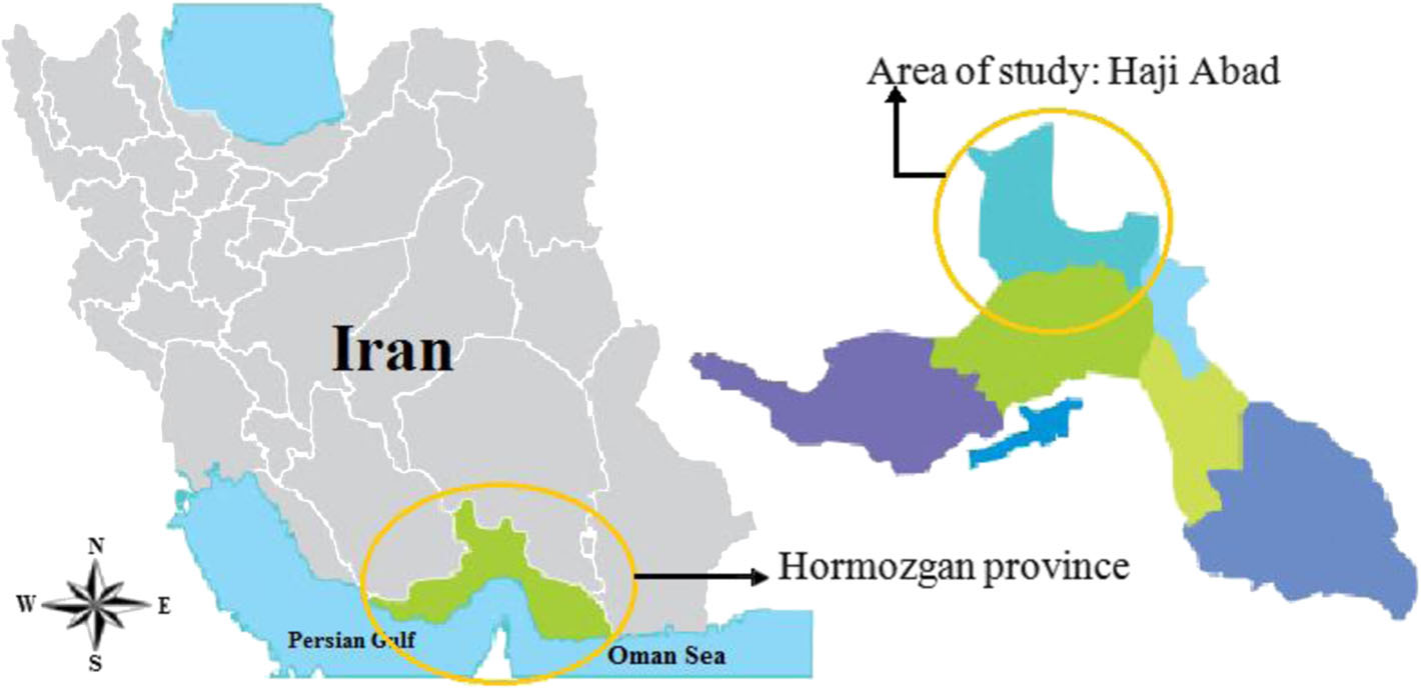
Location of the study area in Hormozgan province, south Iran

The study region, Haji-Abad, is in the north of Hormozgan province, south Iran (Fig. 1). This city is located at 28°18′33″N, 55°54′6″E of the equator and based on census of 2011, carried out by the statistical center of Iran (SCI), its total population was approximately 66,000 inhabitants in about 11,000 km^2^ of area. About 43% of its inhabitants live in urban areas. The greater distance between villages is up to 120 km. This province has tropical climate with low temperatures of about −3.6 °C during winter (December-January) and about 46.6 °C during summer (July-June) whereas annual rainfall is about 160 mm. The average annual relative humidity, wind speed, and sunlight hours are 40%, 17 m/s, and 3466 h, respectively [13]. There are seven species of scorpions that can be found in this city which that belong to the families Buthidae and Liochelidae [13].

### Data acquisition

All scorpion sting data (*n* = 853) collected from May 2012 to July 2016 in ten rural health centers, two healthcare stations and Haji-Abad central hospital of Hormozgan University of Medical Sciences (HUMS) were retrospectively analyzed. Information from the available documents included demographic and epidemiological characteristics of scorpion stings along with climate records obtained from the Bureau of Meteorology Station of Haji-Abad. The only exclusion criterion was the deficit in data. This study was a retrospective cross-sectional one.

Demographic and epidemiologic variables included age, gender, victim’s job, region (urban/rural), date of the sting, location (indoor/outdoor), affected limb (trunk, hand, head or neck, and leg), time of the event (12 p.m.-6 a.m., 6 a.m.-12 a.m., 12 a.m.-6 p.m. or and 6 p.m.-12 p.m.), the elapsed time between sting and treatment (< 3 h, 3-6 h, > 6 h), history of sting (scorpion, snake, and none).

The clinical symptoms were local (redness around the sting site, local pain, numbness in the limb or body, and severe muscular pain) or systemic (signs of sympathetic/parasympathetic nervous systems, and central nervous system).

The climatic factors – monthly averages of temperature (T) in °C, rainfall (R) in mm, relative humidity (RH) in percentage (%), wind velocity (WV) in m/s, and sunlight hours (SH) – were assessed as independent factors in the Pearson correlation statistics and multiple regression model.

### Statistical analysis

Descriptive statistics (number of frequency and percentage) and chi-square test were used to present the epidemiologic data in the current study. Mixed seasonal ARMA method was implemented to describe the behavior of data over time. To select the best model, root mean square error (RMSE) values obtained from the residuals of the model fitting, application of the modified Box-Pierce test, and other diagnostic measures including autocorrelation (ACF) and partial autocorrelation (PACF) functions were calculated. Among the candidate models, the one that consistently had the smallest value of RMSE and also satisfied most diagnostic measures was selected as the best fitting model [15–17].

The Pearson correlation statistics (r) was also applied to determine any significant relation between the climatic factors and the monthly activity of scorpions [13]. The (weighted) multiple regression analysis was also used to generate a formula for describing and predicting the average amount of antivenom that would be required per month to treat victims of scorpion stings [18, 19]. All the statistical analyses were carried out using Minitab software, version 17.1.0 and SPSS software, version 16.0. *p* < 0.05 was considered significant.

## Results

### Demographic and epidemiologic findings

During the study period, from May 2012 to July 2016, a total of 853 patients were registered in the Haji-Abad health centers, south Iran. The incidence of scorpion sting cases was 13 per 1000 people during the 51 months of the study period. Of all patients, 384 (45%) were females. The mean (± SD) age of stung victims was 30.1 (± 19.6) years (range: 1-90 years) and the most commonly involved age group was from 20 to 29 years (*n* = 186, 21.8%). From 853 scorpion sting cases, 30.2% (*n* = 258) were from urban areas and the rest from rural areas. Among urban victims, 147 (57%) were females. Most (*n* = 467, 54.7%) patients were unemployed and farm workers followed by housewives (*n* = 257, 30.2%) (Table 2).

**Table 2:** Demographic and epidemiological characteristics of patients (*n* = 853)

	Subgroup	Male (*n* = 469)	Female (*n* = 384)	Total (%)
		Number (%)	Number (%)	
Demographic variables				
Region of occurrence	Rural	322 (64.9)	273 (71.1)	595 (69.8)
	Urban	147 (35.1)	111 (28.9)	258 (30.2)
Age of victims (years)	1–9	93 (19.8)	75 (19.5)	168 (19.7)
	10–19	58 (12.4)	53 (13.8)	111 (13.0)
	20–29	112 (23.9)	74 (19.3)	186 (21.8)
	30–39	75 (16.0)	53 (13.8)	128 (15.0)
	40–49	42 (9.0)	51 (13.3)	93 (10.9)
	>50	89 (18.9)	78 (20.3)	167 (19.6)
Profession of the victim	Housewife	0 (0.0)	257 (66.9)	257 (30.2)
	Student	55 (11.7)	40 (10.4)	95 (11.1)
	Employee	28 (6.0)	6 (1.6)	34 (4.0)
	Unemployed and farmers	386 (82.3)	81 (21.1)	467 (54.7)
Epidemiological variables				
Anatomical region of the sting	Hand	225 (48.0)	146 (38.0)	371 (43.5)
	Leg	160 (34.1)	161 (41.9)	321 (37.7)
	Trunk	58 (12.4)	53 (13.8)	111 (13.0)
	Neck	26 (5.5)	24 (6.3)	50 (5.8)
Previous history of venomous sting/bite	Snake bite	11 (2.3)	3 (0.8)	14 (1.6)
	Scorpion sting	204 (43.5)	167 (43.5)	371 (43.4)
	Unknown	254 (54.2)	214 (55.7)	468 (55.0)
Time (h) elapsed between accident and medical assistance	< 3	353 (75.3)	278 (72.4)	631 (74.1)
	3–6	61 (13.0)	53 (13.8)	114 (13.3)
	> 6	55 (11.7)	53 (13.8)	108 (12.6)
Location of sting occurrence	Indoors	210 (44.8)	248 (64.6)	458 (53.7)
	Outdoors	176 (37.5)	87 (22.7)	263 (30.9)
	Unknown	83 (17.7)	49 (12.7)	132 (15.4)
Time of sting	0–6	132 (28.1)	117 (30.5)	249 (29.2)
	6–12	93 (19.8)	70 (18.2)	163 (19.1)
	12–18	60 (12.8)	48 (12.5)	108 (12.6)
	18–24	81 (17.4)	69 (18.0)	150 (17.6)
	Unknown	103 (21.9)	80 (20.8)	183 (21.5)
Color of scorpion	Yellow	363 (77.4)	300 (78.1)	663 (77.8)
	Black	57 (12.2)	46 (12.0)	103 (12.1)
	Unknown	49 (10.4)	38 (9.9)	87 (10.1)

The majority of the stings (*n* = 663, 77.8%) were provoked by the yellow scorpion *Odontobuthus doriae,* followed by the black scorpion *Androctonus crassicauda* (*n* = 103, 12.1%). The number of stings by yellow scorpions was six times higher than that of black scorpions. Yellow scorpion stings were significantly more frequent than others among rural and urban victims (*χ* ^2^ *=* 6.17, *p* = 0.04). Most scorpion stings occurred indoors (*n* = 458, 53.7%) and between midnight and 6 a.m. (*n* = 249, 29.2%). In addition, the chi-square test indicated that the place where the stings occurred (indoors/outdoors) significantly varied according to the gender of the patient (*χ* ^2^ *=* 35.3, *p* < 0.001). The number of scorpion stings was significantly different between roofed and unroofed places (*χ* ^2^ *=* 141.7, *p* < 0.001). Results of this research revealed that most cases of scorpionism (*n* = 631, 74.1%) were referred to the clinic less than three hours after the events (Table 2).

More than one third of the stings were on hands (*n* = 371, 43.5%) followed by legs (*n* = 321, 37.7%), trunks (*n* = 111, 13%), and necks (*n* = 50, 5.8%). Nearly half of the victims (*n* = 371, 43.4%) had a previous history of being stung by scorpion and only a few (*n* = 14, 1.6%) of them had a history of snakebite (Table 2).

**Fig. 2:**
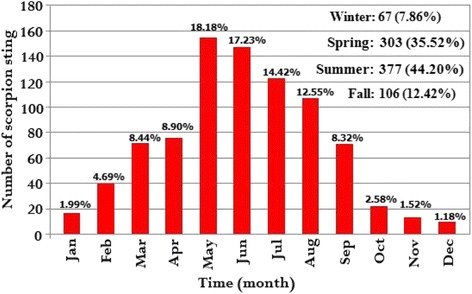
Distribution of scorpion sting cases based on various months and seasons in the city of Haji-Abad, from 2012 to 2016 (*n* = 853)

**Fig. 3:**
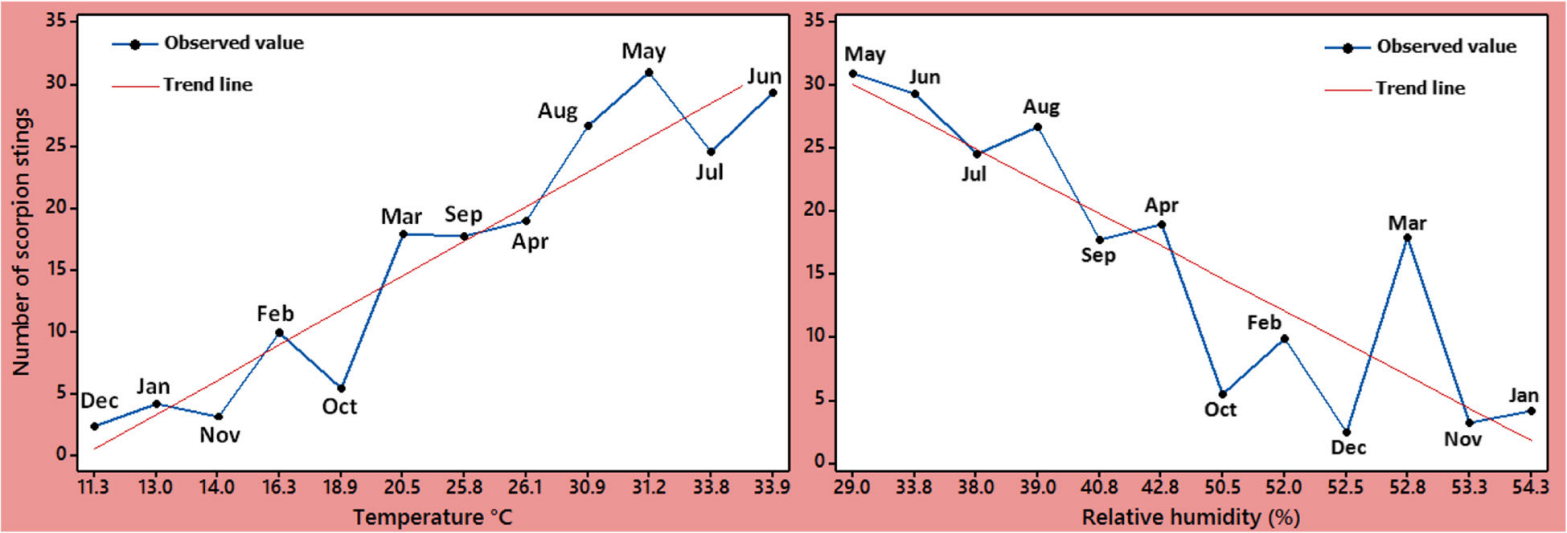
Distribution of average of scorpion sting cases with respect to average temperature (°C) and relative humidity (%) in different months of the years 2012 to 2016

The highest number of scorpion stings was registered in 2014, with 253, cases and the lowest was in 2015, with 173 cases. Most stings (*n* = 377, 44.2%) happened in the summer whereas a small portion of them occurred in the winter (*n* = 67, 7.86%). The lowest (*n* = 10, 1.18%) and the highest (*n* = 155, 18.18%) number of stings were reported in December and May, respectively (Figs. 2 and 3).

### Clinical data

As displayed in Table 3, most patients (*n* = 560, 65.6%) had pain on their stung site. Redness around the stung area was seen in 285 patients (33.4%), but only 21 (2.5%) and 30 (3.5%) of them had numbness in limbs or bodies and severe muscle pains, respectively.

**Table 3:** Description of clinical signs and symptoms of patients stung by scorpions (*n* = 853)

Signs and Symptoms			Males (55%)	Females (45%)	Total (%)
			Number (%)	Number (%)	
Local symptoms	Redness around the sting site	Yes	150 (32.0)	135 (35.2)	285 (33.4)
		No	319 (68.0)	249 (64.8)	568 (66.6)
	Local pain	Yes	308 (65.7)	252 (65.6)	560 (65.6)
		No	161 (34.3)	132 (34.4)	293 (34.4)
	Numbness in limb or body	Yes	11 (2.3)	10 (2.6)	21 (2.5)
		No	458 (97.7)	374 (97.4)	832 (97.5)
	Severe muscle pain	Yes	17 (3.6)	13 (3.4)	30 (3.5)
		No	452 (96.4)	371 (96.6)	823 (96.5)
Systemic symptoms	Signs of sympathetic nervous system	Yes	15 (3.2)	10 (2.6)	25 (3.0)
		No	454 (96.8)	374 (97.4)	828 (97.0)
	Signs of parasympathetic nervous system	Yes	7 (1.5)	5 (1.3)	12 (1.4)
		No	462 (98.5)	379 (98.7)	841 (98.6)
	Signs of central system	Yes	14 (3.0)	9 (2.3)	23 (2.6)
		No	455 (97.0)	375 (97.7)	830 (97.4)

In addition, of all 853 scorpionism victims, only 7% reported systemic symptoms. Most cases were related to the signs of the sympathetic (*n* = 25, 3%) followed by those of the central (*n* = 23, 2.6%), and parasympathetic nervous system (*n* = 12, 1.4%) in stung patients (Table 3).

### Association between climate and scorpion sting cases with Pearson correlation

Biologically, a significant correlation coefficient (r) presents a positive linear relationship if *r* > 0.6. As shown in Table 4, the correlation coefficients (r) between scorpion sting cases and climate factors were considered significant (*p* < 0.05), except for WV (*p* = 0.520). Significant direct correlations were observed between scorpion sting cases and each of T (*r* = 0.708, *p* < 0.001) and SH (*r* = 0.525, *p* < 0.001). Significant negative correlations between the activity of scorpions and each of RH (*r* = −0.728, *p* < 0.001) and R (*r* = −0.335, *p* = 0.015) were also noted (Table 4).

**Table 4:** Correlation coefficients (r) of scorpion stings and the averages of monthly climate factors

Factors	Min.	Max.	Range	Mean	S.D.	r	*p* value
Temperature	9.6	34.4	24.8	23.7	8.2	0.708	<0.001*
Wind velocity	8.0	28.0	20.0	17.2	4.6	−0.091	0.520
Sunlight hours	214.1	383.9	169.8	295.8	40.9	0.525	<0.001*
Relative humidity	19.0	58.0	39.0	44.2	9.7	−0.728	<0.001*
Precipitation	0.0	115.4	115.4	14.9	23.6	−0.335	0.015*

*Min.* minimum, *max.* maximum; *S.D.* standard deviation

*p value <0.05 is significant

### Regression analysis of the study population data

Multiple regression analyses results – when scorpion sting cases were selected as dependent factors, and monthly averages of R, SH, T, RH, and WV as independent factors – are reported in Table 5. As revealed from the unweighted analysis, all the variance inflation factors (VIF) of each covariate are less than 4.4, under the suggested threshold value of 10, and indicating that the co-linearity between the predictive covariates is negligible.

**Table 5:** Factors associated with monthly scorpion sting distribution for the study population via regression analyses per year (scorpion sting rate is response variable)

Model	Factor	$\widehat{b}$	S.E. ($\widehat{b}$)	T value	*p* value	VIF
Unweighted regression	Temperature	0.774	0.315	2.46	0.018*	4.03
	Wind velocity	0.057	0.315	0.18	0.858	1.29
	Sunlight hours	−0.080	0.057	−1.39	0.171	3.30
	Relative humidity	−0.788	0.278	−2.83	0.007*	4.36
	Rainfall	0.020	0.068	0.29	0.773	1.55
	Constant	55.500	22.600	2.46	0.018*	–
	R-squared = 59.47%, Adjusted R-squared = 55.06%, S = 9.22					
Weighted regression	Temperature	1.002	0.182	5.49	<0.001*	3.45
	Wind velocity	0.332	0.189	1.76	0.086	2.00
	Sunlight hours	−0.045	0.031	−1.46	0.152	3.57
	Relative humidity	−0.656	0.196	−3.35	0.002*	3.44
	Rainfall	0.045	0.031	1.46	0.151	3.16
	Constant	29.5	14.700	2.00	0.051	–
	R-squared = 79.00%, Adjusted R-squared = 76.72%, S = 1.27					

$\widehat{\boldsymbol{b}}$ coefficient, *S.E. (* $\widehat{\boldsymbol{b}}$ *)* standard error of coefficient, *VIF* variance inflation factor

*p value <0.05 is significant

The normal probability plot of the residuals did not indicate any reason for departures from normality assumption (unpublished graph) and it depicts a suitable validation for the assessment of the regression model. In addition, the plot of the residuals versus the fitted values for the unweighted multiple regression analysis (unpublished graph) revealed possible non-constant variance because of the existence of a megaphone pattern. Therefore, weighted multiple regression to overcome the problem of non-constant variance was applied. The observed pattern of residual plot for the weighted multiple regression analysis is approximately similar to a horizontal bar, which states the lack of a violation of the normality assumption of the error terms and, also, supports the adequacy of the model.

Significant climate factors associated with activity of scorpions are listed in Table 5. The factors T and RH were statistically significant ($\widehat{b}$ = 1.002 and −0.656; *p* < 0.001 and *p* = 0.002, respectively). By holding other factors in the model constant, any increment in T and decrease in RH would culminate in a growth of scorpion sting cases. The stated findings also corroborate the tendency displayed in Figs. 2 and 3, in which the majority of scorpion stings happened in the warmer months and the trend line of RH has downward slope.

The following equation was employed to predict the monthly number of scorpion sting cases:

$$ \mathrm{Scorpion}\  \mathrm{sting}\  \mathrm{cases}=1.002\ \mathrm{T}+0.332\ \mathrm{WV}\hbox{--} 0.045\ \mathrm{SH}\hbox{--} 0.656\ \mathrm{R}\mathrm{H}+0.045\ \mathrm{R}+29.5 $$

**Fig. 4:**
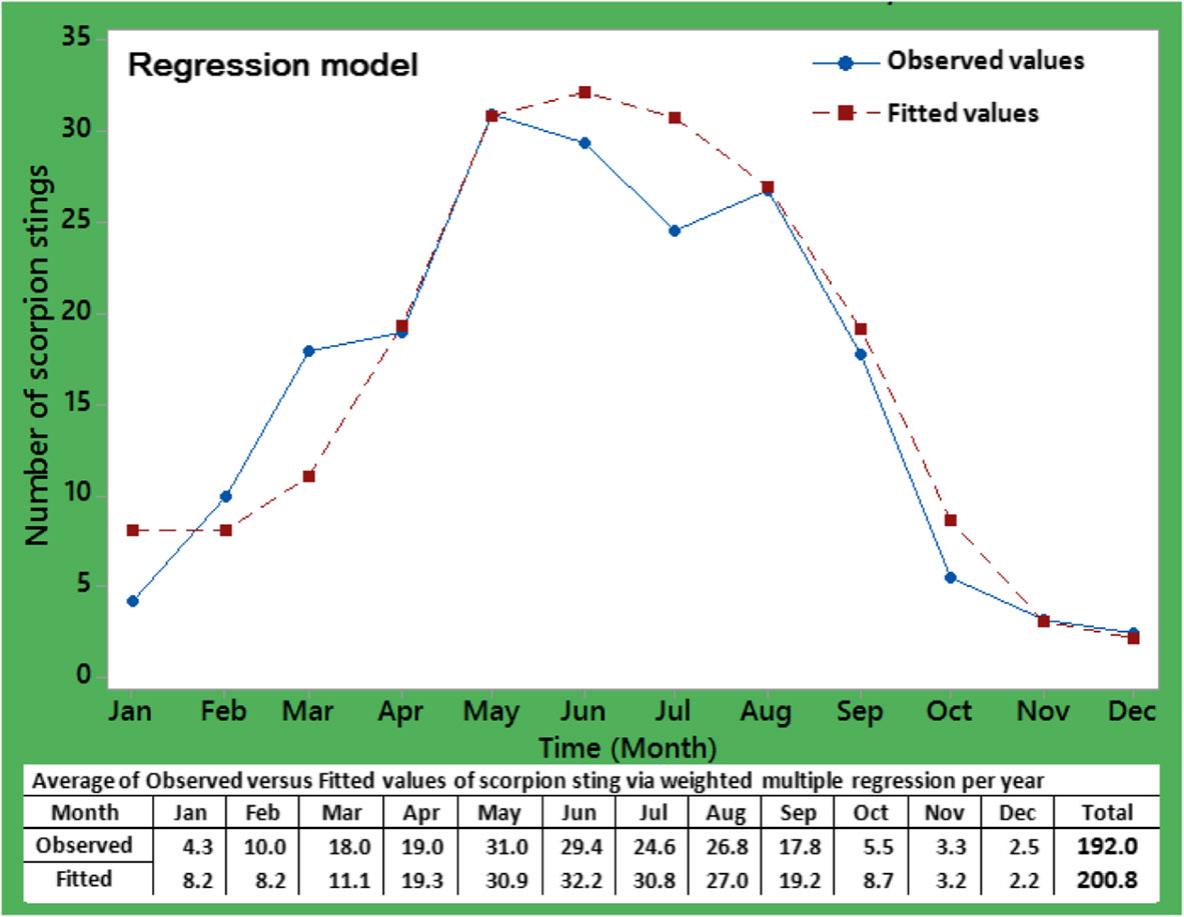
Plot of average of the observed and fitted values for scorpion sting cases between the years 2012 and 2016 using weighted regression model

The equation is relevant since it indicates an estimate of the number of antivenom vials that should be available per year. Weighted R-squared was 0.79, indicating that 79% of the variation in scorpion sting cases can be explained jointly by the five selected climate factors. The remaining variation, about 21%, in dependent variable can be illustrated using residuals or other factors other than the elected climate factors as well as socioeconomic factors. The plot of the observed data and the fitted values over the study interval are shown in Fig. 4.

### Time series process to detect monthly trend of scorpion sting cases

**Fig. 5:**
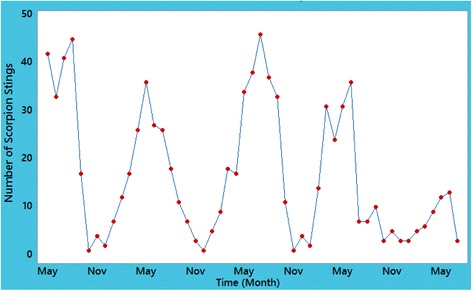
Time series curve of scorpion sting cases from May 2012 to July 2016

**Fig. 6:**
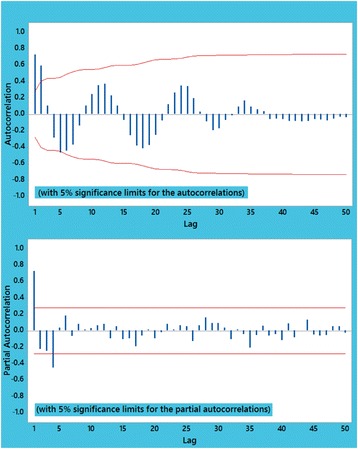
Plot of autocorrelation (ACF) and partial autocorrelation (PACF) functions for scorpion sting cases

The time series plot of scorpion sting data in Fig. 5 contains no special pattern, ascending or descending, throughout the studied time period and there is a random behavior over time. Both plotted ACF and PACF functions in Fig. 6 can specify the order of time series processes. These plots revealed that *p* = 1 and q = 2; thus, ARMA (1, 2) was fitted as a proposed process. However, the first order of MA, i.e. MA (1), was not statistically significant (*p* = 0.384) and eventually, ARMA (1, 1) was applied as another suggested process. Although all of the coefficients in the ARMA (1, 1) model were significant, modified Box-Pierce test stated that this process was not statistically suitable and good enough (*p* < 0.05).

A seasonal trend was observed when checking associated residual plot of the data (unpublished graph). Various combinations for mixed seasonal ARMA model, ARMA (p, q) × (P, Q)_*h*_ (p, q) × (P, Q) at lag h, on the current data set were done and it was observed that ARMA (1, 1) × (0, 1) at lag 12 process was the best fitted; therefore, it was applied as an optimal model (Table 6). Among three processes in Table 6, RMSE for this seasonal process was calculated to be equal to 8.09, which is lower than the corresponding RMSE value for the other processes, indicating a better fit for ARMA (1, 1) × (0, 1) at lag 12. The modified Box-Pierce test for this optimal model shows that the model is statistically detected well and no significant statistical difference exists between the observed and the fitted values by model.

**Table 6:** Three candidate processes for the study population via time series models per year

Model				
ARMA (1, 1)	$\widehat{b}$	S.E. ($\widehat{b}$)	*p* value	RMSE
Constant	5.90	1.67	0.001*	8.68
AR (1)	0.66	0.13	< 0.001*	
MA (1)	−0.37	0.16	0.025*	
Modified Box-Pierce test	Lag	Chi-square	df	*p* value
	12	24.0	9	0.004*
	24	33.9	21	0.037*
ARMA(1, 2)	$\widehat{b}$	S.E. ($\hat{b}$)	*p* value	RMSE
Constant	0.72	0.07	< 0.001*	10.06
AR (1)	0.96	0.25	< 0.001*	
MA(1)	0.25	0.29	0.384	
MA (2)	0.71	0.20	0.001*	
Modified Box-Pierce test	Lag	Chi-square	df	*p* value
	12	40.7	8	< 0.001*
	24	80.7	20	< 0.001*
ARMA (1, 1) × (0, 1)_12_	$\hat{b}$	S.E. ($\widehat{b}$)	*p* value	RMSE
Constant	6.42	2.21	0.006*	8.09
AR (1)	0.63	0.14	< 0.001*	
MA (1)	−0.38	0.16	0.026*	
SMA (1)	−0.41	0.14	0.005*	
Modified Box-Pierce test	Lag	Chi-square	df	*p* value
	12	13.2	8	0.104
	24	19.9	20	0.466

*ARMA* auto-regressive moving average, *ARMA* (*p*, *q*) × (*P*, *Q*)_*h*_ mixed seasonal ARMA, $\widehat{\boldsymbol{b}}$ coefficient, *df* degree of freedom, *S.E. (* $\widehat{\boldsymbol{b}}$ *)* standard error of coefficient, *RMSE* root mean square error

*p value <0.05 is significant

**Fig. 7:**
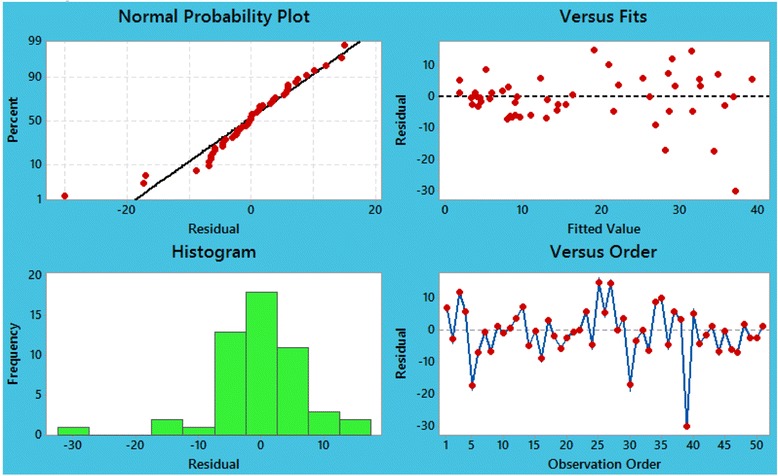
Plot of residual versus fitted values, histogram of residuals, normal probability plot, and plot of residual versus ordered times for scorpion sting cases in fitting mixed seasonal ARMA, ARMA (1, 1) × (0, 1) model at lag 12

**Fig. 8:**
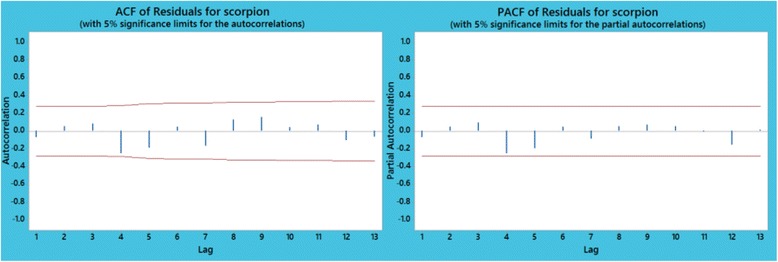
Plot of autocorrelation (ACF) and partial autocorrelation (PACF) of residuals for scorpion sting data set

As another diagnostic check, the 4-in-1 residual plots were depicted in Fig. 7. These plots demonstrated that the fitting was indeed quite good and confirmed aptness of the suggested model. The adequacy of the proposed model was also proven in Fig. 8. In this figure, plots of ACF and PACF of residuals state that partial autocorrelations and autocorrelations are near zero, which confirms that the residuals were not significant at all lags.

**Fig. 9:**
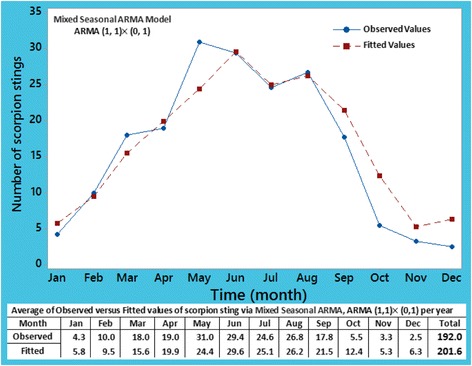
Plot of average of the observed and fitted values for scorpion sting cases in the years 2012 to 2016 using mixed seasonal ARMA, ARMA (1, 1) × (0, 1) at lag 12 model

Finally, plot of the observed data and the fitted values simultaneously over the study period are presented in Fig. 9. It appears that fitted values smooth out the highs and lows in the data, demonstrating that the fitted values are a suitable and a good estimator of observed values.

## Discussion

The main findings in this study showed that scorpion stings due to *Odontobuthus doriae* were significantly higher than those provoked by other species among urban and rural patients (*p* = 0.04). The highest frequency of scorpion stings occurred mostly in rural areas (Table 2). These outcomes were confirmed by several other studies [20–24]. However, some reports have demonstrated that stings occurred more often in urban regions [25]. Due to the lack of safe standard houses and proximity to the living places of scorpions, the entry of scorpions into human dwellings in most villages is easier; it is, thus, expected that stings in rural areas are more common than in urban areas.

The present study data indicated that the age group with the highest frequency of scorpion stings was the 20-29-year old group (Table 2). These results were consistent with the studies performed by other groups [26]. In Turkey, researchers stated that scorpion stings frequently (54.1%) affected children aged 9-15 years when compared to other age groups [27]. The high frequency of scorpion stings among young people is mainly associated with their outdoor activities in farms and gardens, which expose them to stings. Farming, irrigation and lack of sufficient artificial light could be implicated in their high exposure to scorpion stings.

The obtained epidemiological data indicated that nearly 53.7% of stings occurred in roofed places (Table 2), whereas other studies in Brazil [28, 29] and Iran [30] reported that about 90 and 42% of stings occurred indoors, respectively. The chi-square test in the present study demonstrated that the place of being stung (outdoors/indoors) significantly varied according to the gender of the patient (*χ* ^2^ = 35.3, *p* < 0.001). Therefore, in roofed places women were more stung by scorpions than men were; whereas in unroofed places this phenomenon was reversed. This can be due to the fact that in this study area women, unlike men, spend most of their time at home (housewives).

The current results clearly reflected that most patients were stung by scorpions between midnight and 6 a.m. (Table 2), which is mainly due to nocturnal habits of scorpions [30]. Such findings were also corroborated by other studies [31–33]. Farmers and housewives were more at risk of being stung by scorpions, since about 70% of stings were recorded in rural regions and because of the abundant brushwood around the rural houses. These findings were similar to those of other researchers [34].

The time interval between the sting and treatment were less than 3 h for 74.1% of patients (Table 2). Other studies have demonstrated that approximately 96% of the scorpion sting cases were taken to health clinics in less than 3 h [25], and that this high percentage indicates a good awareness of the population on this problem. This elapsed time between the sting and treatment has also been less than 3 h in 69.6% of cases in a study in the western Brazilian Amazon [26, 29]. Nonetheless, in the present study the delay in seeking medical help of 108 patients (12.6%), who received clinical attention 6 h after being stung by scorpions, could indicate that lack of awareness concerning immediate referral to health centers and also inadequate access to health care. Therefore, factors including transportation problems, limited access to health care and delay in clinical examination could prevent treatment at proper time.

Legs and hands were mostly affected by scorpion stings (Table 2). Several studies were consistent with these observations. In most studies, moving parts were at greater risk of being stung in comparison with other parts of body [22]. The likely reason is that many victims do not use appropriate protective tools such as boots and gloves in the farmlands and dooryard gardens where they are active.

The percentage of yellow scorpions (*Odontobuthus doriae)* involved in accidents was more than six-fold that of black scorpions (*Androctonus crassicauda*), 77.8 and 12.1% (Table 2). Numerous studies do not agree with these data [5, 35]. The percentage of black scorpions in other works was much higher than that of yellow scorpions. In other words, the prevalence of yellow scorpion fauna is higher than that of black ones in Haji-Abad.

The activity of scorpions increased from January to the end of May. After that, there is a decrease in sting cases to the end of the year (December). Similar studies in Iran and other parts of the world have confirmed this trend [2, 35, 36]. The results of a study in Saudi Arabia have indicated that most stings (79.2%) occurred from May to October [35]. In addition, in Texas the peak of the scorpion stings occurred from June to September [36]. These differences will likely be due to changes in geographic and abiotic factors. Various reasons are behind this phenomenon. In fact, since scorpions are cold-blooded arthropods, they are more active in warm months and probably these months comprise their reproduction period [23]. Most likely, scorpions enter human dwellings during warm months to catch prey, which causes an increase in their activity [37].

Scorpion sting severity is affected by several variables including scorpion species, climate factors, geographic sites etc. [38]. It should be noted that the present study aimed to examine the factors that influence the activity of scorpions and to forecast the necessary amount of polyvalent antivenom using mixed seasonal ARMA method.

According to the results of regression analysis in Table 5, the two significant factors affect the outcome of scorpion sting cases are monthly averages of T and RH. Consequently, any increase in these variables influence the activity of scorpions, which is confirmed by Figs. 2 and 3 and also previous studies on scorpion envenomation [23, 39]. Being cold-blooded arthropods, scorpions are affected by the RH and T of the environment. This is the reason why in cold climates the number of scorpions becomes small, whereas numerous scorpions species are found in tropical and subtropical regions [23].

The present collected data indicated that scorpion sting cases presented a similar structure during the study period of 51 months. Therefore, the use of a time series model was a suitable approach to characterize the monthly trend of data [15, 16]. After attempts to fit various time series models, it was found that the mixed seasonal ARMA (1, 1) × (0, 1)_12_ process was suitable to use in scorpion stings data from south Iran. Recently, several studies have investigated this trend of the activity of scorpions over time. These surveys undertook ARMA (2, 1) and SARIMA (5, 1, 0) × (0, 1, 1) at lag 12 models in their analyses to describe the behavior of data over time [23, 39].

A comparison between both plots in Figs. 4 and 9, and in Tables 5 and 6, reveals that in anticipation of future scorpion sting cases, the efficiency of mixed seasonal ARMA (1, 1) × (0, 1) at lag 12 is almost identical to the regression analysis. Therefore, to predict future cases of scorpionism (thus, the monthly average amount of required antivenom) around Haji Abad and similar tropical regions, the use of both methods can approximately have the same results.

There were several limitations to the present study. Due to its design type, the research was limited by the inaccessibility of some clinical data and laboratory data including blood and urine analysis. Other areas must be added for comparison. Moreover, additional antecedents must be taken into account for in-depth insight of the under study form.

## Conclusions

Most studies had applied only the descriptive approach in their analyses. In the present work, the analysis of time series to determine the behavior of data over time and to predict the monthly average number of required antivenom vials were also employed. Mixed seasonal ARMA was a useful tool to monitor the cases of scorpion stings.

Therefore, utmost precaution should be adopted between midnight and 6 a.m. and in warmer months by health care centers to provide necessary aid for scorpion sting victims. Additionally, young rural housewives and farmers must be offered educational activities and knowledge on good health measures around Haji-Abad and other tropical areas.

## Abbreviations

ACF: Autocorrelation function
AR: Autoregressive
ARMA: Autoregressive moving average
HUMS: Hormozgan University of Medical Sciences
MA: Moving average
PACF: Partial autocorrelation function
R: Rainfall
RH: Relative humidity
RMSE: Root mean square error
SH: Sunlight hours
T: Temperature
VIF: Variance inflation factors
WV: Wind velocity
